# Range Expansion and the Origin of USA300 North American Epidemic Methicillin-Resistant *Staphylococcus aureus*

**DOI:** 10.1128/mBio.02016-17

**Published:** 2018-01-02

**Authors:** Lavanya Challagundla, Xiao Luo, Isabella A. Tickler, Xavier Didelot, David C. Coleman, Anna C. Shore, Geoffrey W. Coombs, Daniel O. Sordelli, Eric L. Brown, Robert Skov, Anders Rhod Larsen, Jinnethe Reyes, Iraida E. Robledo, Guillermo J. Vazquez, Raul Rivera, Paul D. Fey, Kurt Stevenson, Shu-Hua Wang, Barry N. Kreiswirth, Jose R. Mediavilla, Cesar A. Arias, Paul J. Planet, Rathel L. Nolan, Fred C. Tenover, Richard V. Goering, D. Ashley Robinson

**Affiliations:** a Department of Microbiology and Immunology, University of Mississippi Medical Center, Jackson, Mississippi, USA; b Cepheid, Sunnyvale, California, USA; c Department of Infectious Disease Epidemiology, Imperial College London, London, United Kingdom; d Microbiology Research Unit, Dublin Dental University Hospital, University of Dublin, Trinity College Dublin, Dublin, Ireland; e School of Veterinary and Life Sciences, Murdoch University, Perth, Australia; f PathWest Laboratory Medicine—WA, Fiona Stanley Hospital, Perth, Australia; g Instituto de Investigaciones en Microbiología y Parasitología Médica, Universidad de Buenos Aires and CONICET, Buenos Aires, Argentina; h Center for Infectious Diseases, Division of Epidemiology, Human Genetics and Environmental Sciences, University of Texas Health Science Center, Houston, Texas, USA; i Statens Serum Institut, Copenhagen, Denmark; j Molecular Genetics and Antimicrobial Resistance Unit, International Center for Microbial Genomics, Universidad El Bosque, Bogota, Colombia; k Department of Microbiology and Medical Zoology, University of Puerto Rico, San Juan, Puerto Rico; l Department of Pathology and Microbiology, University of Nebraska Medical Center, Omaha, Nebraska, USA; m Department of Internal Medicine, Division of Infectious Diseases, The Ohio State University, Columbus, Ohio, USA; n Public Health Research Institute of New Jersey Medical School, Rutgers University, Newark, New Jersey, USA; o Center for Antimicrobial Resistance and Microbial Genomics, University of Texas McGovern School of Medicine at Houston, Houston, Texas, USA; p Children’s Hospital of Philadelphia, University of Pennsylvania, Philadelphia, Pennsylvania, USA; q Department of Internal Medicine, Division of Infectious Diseases, University of Mississippi Medical Center, Jackson, Mississippi, USA; r Department of Medical Microbiology and Immunology, Creighton University, Omaha, Nebraska, USA; University of California, Irvine

**Keywords:** epidemics, fluoroquinolones, founder effects, genetic drift, population genetics, range expansion

## Abstract

The USA300 North American epidemic (USA300-NAE) clone of methicillin-resistant *Staphylococcus aureus* has caused a wave of severe skin and soft tissue infections in the United States since it emerged in the early 2000s, but its geographic origin is obscure. Here we use the population genomic signatures expected from the serial founder effects of a geographic range expansion to infer the origin of USA300-NAE and identify polymorphisms associated with its spread. Genome sequences from 357 isolates from 22 U.S. states and territories and seven other countries are compared. We observe two significant signatures of range expansion, including decreases in genetic diversity and increases in derived allele frequency with geographic distance from the Pennsylvania region. These signatures account for approximately half of the core nucleotide variation of this clone, occur genome wide, and are robust to heterogeneity in temporal sampling of isolates, human population density, and recombination detection methods. The potential for positive selection of a *gyrA* fluoroquinolone resistance allele and several intergenic regions, along with a 2.4 times higher recombination rate in a resistant subclade, is noted. These results are the first to show a pattern of genetic variation that is consistent with a range expansion of an epidemic bacterial clone, and they highlight a rarely considered but potentially common mechanism by which genetic drift may profoundly influence bacterial genetic variation.

## IMPORTANCE

The process of geographic spread of an origin population by a series of smaller populations can result in distinctive patterns of genetic variation. We detect these patterns for the first time with an epidemic bacterial clone and use them to uncover the clone’s geographic origin and variants associated with its spread. We study the USA300 clone of methicillin-resistant *Staphylococcus aureus*, which was first noticed in the early 2000s and subsequently became the leading cause of skin and soft tissue infections in the United States. The eastern United States is the most likely origin of epidemic USA300. Relatively few variants, which include an antibiotic resistance mutation, have persisted during this clone’s spread. Our study suggests that an early chapter in the genetic history of this epidemic bacterial clone was greatly influenced by random subsampling of isolates during the clone’s geographic spread.

## INTRODUCTION

The first known cases of infection caused by the USA300 clone of methicillin-resistant *Staphylococcus aureus* (MRSA) were from outbreaks of skin and soft tissue infections (SSTIs) in a prison in Mississippi in November 1999 and in a sports team in Pennsylvania in September 2000 (1–3). By 2002, SSTIs caused by USA300 had occurred across the United States (3, 4). By 2008, this clone was the leading cause of SSTIs seen in emergency departments and was an increasing cause of health care-associated bloodstream infections (5–8). As of 2012, USA300 was the predominant MRSA from all infection sites reported in multistate surveillance in the United States (9, 10). A notable feature of USA300 infections, besides their aggressive course, is that healthy young people without risk factors for health care-associated MRSA infections are often affected. These types of infections were called community-associated MRSA infections and had not been observed in such a volume prior to the emergence of USA300 (11).

The USA300 clone was originally defined by a unique pulsed-field gel electrophoresis (PFGE) pattern (12) that occurs with other traits, including multilocus sequence type 8, *spa* type t008, the presence of the staphylococcal chromosomal cassette *mec* (SCC*mec*) type IV element and the arginine catabolic mobile element (ACME), and the ability to express the Panton-Valentine leucocidin (PVL) (2, 13). In 2005, a PFGE profile and SCC*mec* variant of USA300 that lacked ACME was identified in northern South America with clinical and epidemiological characteristics similar to those of the North American clone (14, 15). Phylogenomic analysis demonstrated that the North American epidemic (NAE) and South American epidemic (SAE) clones are monophyletic sister clades that have caused parallel epidemics in the Americas (16). Export of both clones to other continents has occurred sporadically (17–19).

Transmission of USA300-NAE is primarily through close person-to-person contact such as within households and among other populations living under crowded conditions and sharing personal items (3, 20, 21). Only limited phylogenomic clustering of USA300-NAE isolates by neighborhoods, hospitals, and cities has been observed, suggesting migration between these populations (20–23). Further use of a phylogenomic approach to reveal the origin and spread of USA300-NAE in the United States has been hindered by sparse geographic sampling and the clone’s poorly resolved, star-like phylogeny. Thus, despite the ongoing public health significance of USA300-NAE, the fundamental question of where it originated remains unanswered.

Here we infer the geographic origin of USA300-NAE through the use of genomic signatures that are expected to occur when an origin population expands its geographic range by a series of smaller populations (24). These smaller populations represent the expansion front and are subject to potentially strong genetic drift arising from their serial founding (i.e., sequential bottlenecking). Consequently, these smaller populations are expected to have decreased genetic diversity and an increased derived allele frequency, on average, with distance from the origin population (25–27). While all epidemic bacterial clones will expand their geographic range to some degree, the power to detect these genomic signatures will depend on many demographic factors, including the expansion time, bottleneck sizes, and rates of migration between populations (28–30). Results from several studies indicate that the most recent common ancestor of USA300-NAE existed at least 7 years prior to the first report of this clone in the literature (16, 19–21), which might have allowed genetic diversity to build near its origin population prior to the spread and establishment of geographically distant populations.

With the resolution provided by genome sequencing, we observe two significant signatures of range expansion of USA300-NAE that independently outline the same region of origin in the eastern United States. We demonstrate that these signatures are genome wide and robust to potential confounders and analysis artifacts. Some unusual polymorphisms accompanying the spread of this clone are also identified, which builds upon prior work (20, 21). One major implication of our study is the realization that genetic drift caused by geographic range expansion may play a large role in shaping the genetic variation of bacteria, even for epidemic clones where a leading role for natural selection may be presumed.

## RESULTS

### Phylogenomic delineation of the USA300-NAE clade.

**FIG 1:**
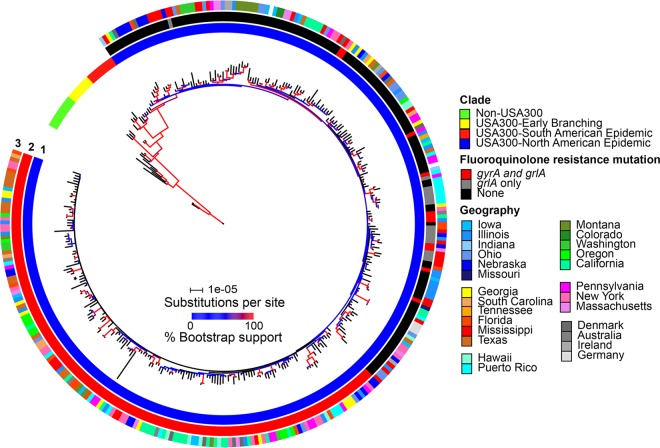
ML phylogeny of USA300 with branch lengths corrected for recombination by CFML. Branch color indicates bootstrap support of the tipward node. Circle 1 indicates clades. Circle 2 indicates an FQ resistance mutation(s). The asterisk indicates an isolate with a resistance mutation in *gyrA* but not *grlA*. Circle 3 indicates the geographic sources of isolation.

From 357 genome sequences of USA300 and closely related isolates (see Data Set S1 in the supplemental material), a total of 4,109 biallelic single nucleotide polymorphisms (SNPs) and 2.16 Mbp of invariant sites were extracted from the nonrepetitive core genome. A maximum-likelihood (ML) phylogeny with branch lengths corrected for recombinant sequences and a time-stamped Bayesian phylogeny from nonrecombinant sequences showed that 330 isolates were USA300-NAE, 9 were USA300-SAE, and 18 were early-branching USA300 or non-USA300 (Fig. 1, circle 1; Fig. S1 in Text S1). The USA300-NAE, SAE, and NAE+SAE clades each had 100% bootstrap and posterior probability support on these trees. Relationships within the USA300-NAE clade had relatively poor support; 116 (35%) of 329 nodes on the ML phylogeny had >0.7 bootstrap support, and 162 (49%) of 329 nodes on the Bayesian phylogeny had >0.95 posterior probability. Nonetheless, this clade’s very low rate of recombination (ρ per site = 0.00003) indicated that many features of its phylogeny were probably accurate (31). One prominent feature, noted previously (19–21), was the presence of a monophyletic fluoroquinolone (FQ)-resistant subclade, which was defined by resistance mutations in *gyrA* (encoding Ser84Leu) and *grlA* (encoding Ser80Tyr) and represented 157 (48%) of 330 USA300-NAE isolates (Fig. 1, circle 2; Fig. S1 in Text S1). Only 9 (5%) of 173 USA300-NAE isolates outside this subclade had these resistance alleles. On the basis of an estimated mutation rate of 6.15 × 10^−7^ substitutions/site/year (95% credibility interval, 5.53 × 10^−7^ to 6.77 × 10^−7^) from the Bayesian analysis, the dates for the most recent common ancestor of our sample of USA300-NAE and its FQ-resistant subclade were estimated to be 1992 (1988 to 1996) and 1998 (1998 to 2000), respectively (Fig. S1 in Text S1); these estimates were in good agreement with previous studies (16, 19–21). A phylogeographic analysis to infer the origin of USA300-NAE was inconclusive and was influenced by oversampling of the Mississippi isolates (see Text S1).

10.1128/mBio.02016-17.2 DATA SET S1  Characteristics of study isolates. Download DATA SET S1, XLSX file, 0.1 MB. Copyright © 2018 Challagundla et al. 2018 Challagundla et al. This content is distributed under the terms of the Creative Commons Attribution 4.0 International license.

10.1128/mBio.02016-17.1 TEXT S1  Supplemental materials and methods, references, figures, and tables. Download TEXT S1, PDF file, 0.7 MB. Copyright © 2018 Challagundla et al. 2018 Challagundla et al. This content is distributed under the terms of the Creative Commons Attribution 4.0 International license.

### Genetic structure of USA300-NAE populations.

The relatively poor phylogenomic resolution within the USA300-NAE clade motivated a population genomics approach. When U.S. states and territories were treated as populations, 18 populations had sample sizes of ≥6 isolates and a total of 2,599 biallelic, nonrecombinant SNPs. The Montana, Hawaii, and Puerto Rico populations were subsequently excluded as genetic or geographic outliers, though their inclusion did not qualitatively alter our conclusions (Fig. S2; Table S1 in Text S1). Tajima’s D was negative for each of the remaining 15 U.S. populations, which indicated an abundance of rare alleles, but statistical significance largely followed sample size (Table 1). Overall patterns of genetic variation were similar among the subset of recent isolates from later in the epidemic (2007 to 2011), which represented 229 (86%) of 265 isolates from these 15 populations (Table S2 in Text S1). Finally, while 1,413 SNPs were nonsynonymous (nSNPs) and 516 were synonymous (sSNPs), the ratio of nSNPs to sSNPs (2.7) was close to the expected value of 2.9 under a simple neutral model (32).

**TABLE 1:** Population genomic summary statistics for 15 populations of USA300-NAE

Population(s)	No. of isolates	Avg pairwise F_ST_^a^	θ_π_^a^	θ_W_^a^	Tajima’s D^a^	*P* (D = 0)	Avg tip-to-tip distance^b^ (10^−5^)	Sum of pairwise Ψ values^c^
All 15	265	0.0345	0.0112	0.1483	−2.9279	0.0034	1.8617	
MS	33	0.0350	0.0085	0.0238	−2.4578	0.0139	1.4748	2.5384
CA	29	0.0471	0.0094	0.0245	−2.4000	0.0164	1.5681	0.8437
IL	27	0.0205	0.0104	0.0331	−2.7086	0.0068	1.6446	0.1011
NY	25	0.0288	0.0119	0.0311	−2.4655	0.0137	1.846	−1.1768
NE	22	0.0431	0.0117	0.0225	−1.9546	0.0506	1.8624	−0.0964
TX	21	0.0266	0.0104	0.0255	−2.4299	0.0151	1.6183	0.0379
OH	17	0.0291	0.0151	0.032	−2.2684	0.0233	2.1435	−2.3722
PA	17	0.0244	0.0124	0.0255	−2.2082	0.0272	1.8791	−1.1698
GA	16	0.0200	0.0133	0.0278	−2.2684	0.0233	1.9802	−1.69
FL	14	0.0197	0.0096	0.0201	−2.3475	0.0189	1.4509	0.7842
IA	11	0.0269	0.0099	0.0156	−1.7511	0.0799	1.5167	0.5411
OR	11	0.0665	0.0095	0.0138	−1.5072	0.1318	1.4318	0.4013
MA	9	0.0238	0.0131	0.0183	−1.4688	0.1419	1.8973	−1.2202
WA	7	0.0643	0.0079	0.0088	−0.5311	0.5953	1.2155	3.2673
SC	6	0.0411	0.0116	0.0133	−0.8322	0.4053	1.6125	−0.7896

a The average pairwise F_ST_, θ_π_; θ_W_, and D values were based on 2,599 biallelic, nonrecombinant SNPs.

b The average tip-to-tip distance reflects branch lengths on an ML tree corrected for recombinant sites.

c The sum of pairwise Ψ values was based on 2,595 biallelic, nonrecombinant SNPs where ancestral and derived alleles were assigned.

Spatial genetic structure was detected in pairwise comparisons of the 15 U.S. populations by the significant positive correlation between genetic differentiation (Hudson’s fixation index [F_ST_]) and geographic distance (great-circle distance) (Mantel *r* = 0.397, *P* = 0.0096, *n* = 105 pairs; Table 2). Of the 19 pairwise comparisons with significant differentiation, 14 (74%) involved the West Coast states California, Oregon, and Washington (Table S3 in Text S1). Among recent isolates, spatial genetic structure was also detected (Table 2) and 12 (60%) of 20 pairwise comparisons with significant differentiation involved the West Coast states (Table S3 in Text S1). Spatial genetic structure was also detected with alternative measurements of genetic differentiation such as Weir and Cockerham’s F_ST_, which was identical to ϕ_ST_ with our data (Table S4 in Text S1). Recent work has shown that spatial patterns of rapidly spreading infectious diseases can sometimes be better informed by the connectedness of populations rather than their physical geographic distance (33, 34). As measurements of population connectedness, we examined total numbers of airline passengers and total migration of residences between U.S. states (see Materials and Methods). Total numbers of airline passengers performed better than total numbers of migrants as a predictor of genetic differentiation, but geographic distance remained the most significant predictor of genetic differentiation, even after correction for total numbers of airline passengers (Table 2). Thus, geographic distance was used for subsequent spatial analysis.

**TABLE 2:** Relationships among genetic differentiation, geographic distance, and connectivity from all pairwise comparisons of 15 populations of USA300-NAE

Test^a^ and predictor of genetic distance	All isolates (2001−2011)		Recent isolates (2007−2011)	
	*r*	*P*	*r*	*P*
Mantel				
Geographic distance	0.397	0.0096	0.355	0.0187
Log total airline passengers	−0.330	0.0732	−0.384	0.0408
Log total migrants	−0.210	0.1656	−0.257	0.1112
Partial Mantel				
Geographic distance accounting for log total airline passengers	0.371	0.0245	0.325	0.0585
Log total airline passengers accounting for geographic distance	−0.297	0.0974	−0.357	0.0630

a The Mantel and partial Mantel tests were performed with 10,000 permutations.

### Evidence for range expansion of USA300-NAE from an eastern U.S. origin.

**FIG 2:**
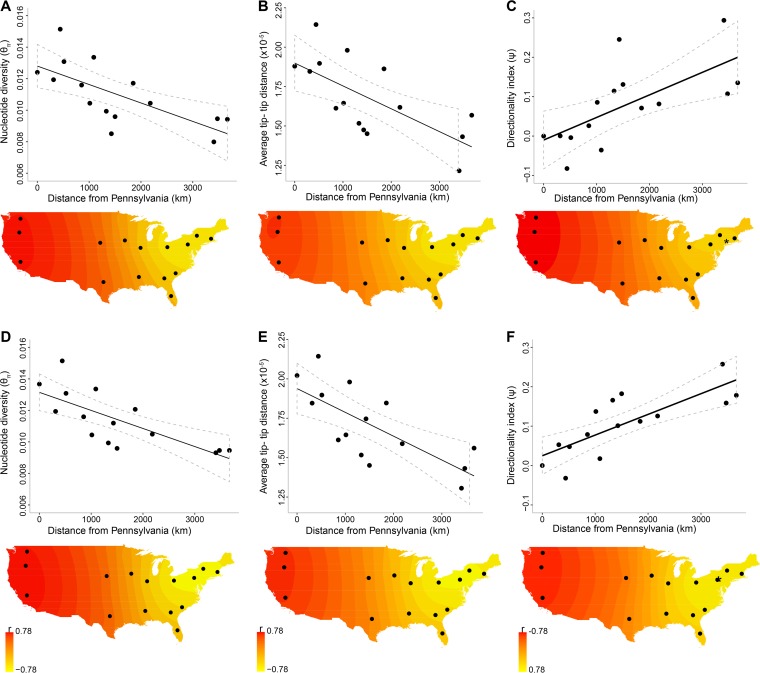
Signatures of range expansion and the origin of USA300-NAE. Panels A to C show the regressions of θ_π_, tip-to-tip distance, and Ψ, respectively, with geographic distance from Pennsylvania, when all isolates (from 2001 to 2011) were used. A solid line indicates the linear regression, and dotted lines indicate the 95% confidence intervals. The maps in panels A to C illustrate the correlations when each of the 15 populations was used as the origin, with interpolation of values between populations. Stronger evidence of origin is shown as yellow. Panels D to F show the results obtained when only recent isolates (from 2007 to 2011) were used. The asterisks on the maps in panels C and F indicate the coordinates of the selected origin when the nonlinear regression of Ψ was used.

The spatial genetic structure described above is referred to as “isolation by distance” and can occur with older populations at migration-drift equilibrium and with nonequilibrium populations that have undergone a recent range expansion (35). To specifically test for a range expansion and to infer the origin of USA300-NAE, we examined the expected signatures of decreased genetic diversity, as measured by θ_π_ and average tip-to-tip distance on the ML tree, and increased derived allele frequency, as measured by the directionality index Ψ, with geographic distance from the origin. The origin was inferred as the population with the most negative or most positive correlation when genetic diversity or derived allele frequency, respectively, was regressed against the geographic distance from that population (see Materials and Methods). Pennsylvania was the most likely origin on the basis of a decrease in θ_π_ (Pearson *r* = −0.697, *P* = 0.0019, the probability of getting a correlation as extreme as the observed correlation under a null hypothesis of panmixia [P_perm_] = 0.024, *n* = 15), a decrease in tip-to-tip distance (Pearson *r* = −0.684, *P* = 0.0025, P_perm_ = 0.031, *n* = 15), and an increase in Ψ by linear regression (Pearson *r* = 0.667, *P* = 0.0033, P_perm_ = 0.029, *n* = 15), but the adjacent population of New York was the origin when the nonlinear regression of Ψ was used (Pearson *r* = 0.686, P_perm_ = 0.029, *n* = 15) (Fig. 2A to C; Table S5 in Text S1). Restriction of the analysis to recent isolates resulted in Pennsylvania as the origin with each signature and with even stronger correlations than with all of the isolates (Fig. 2D to F; Table S6 in Text S1). A clear East-to-West gradient of these correlations was observed (Fig. 2). The West Coast states presented patterns that are the opposite of those expected of an origin; increased genetic diversity and decreased derived allele frequency occurred with distance from the West Coast (Tables S5 and S6 in Text S1).

We also examined whether USA300-NAE genetic diversity could be explained by human population density. Variation in human population density, which is analogous to variation in the carrying capacity of USA300-NAE, as humans are the natural host of this clone, is expected to make the detection of a range expansion more difficult (27, 36). Human population density data for the years 2000 and 2010 showed a significant positive correlation with θ_π_ (year 2000, Pearson *r* = 0.464, *P* = 0.041, *n* = 15; year 2010, Pearson *r* = 0.450, *P* = 0.046, *n* = 15), but not with tip-to-tip distance or Ψ. In a multivariate analysis of the decrease in θ_π_ with the distance from Pennsylvania and accounting for human population density, the range expansion signature remained significant (year 2000, adjusted *r* = 0.652, *P* = 0.014, *n* = 15; year 2010, adjusted *r* = 0.650, *P* = 0.015, *n* = 15) and human population density was not a significant covariate (year 2000, *P* = 0.493; year 2010, *P* = 0.512). In summary, these results show a pattern of USA300-NAE genetic variation that is consistent with a process of geographic range expansion from an eastern U.S. origin that cannot be explained by heterogeneity in the temporal sampling of isolates or by human population density.

### Additional studies of the USA300-NAE range expansion signatures.

**FIG 3:**
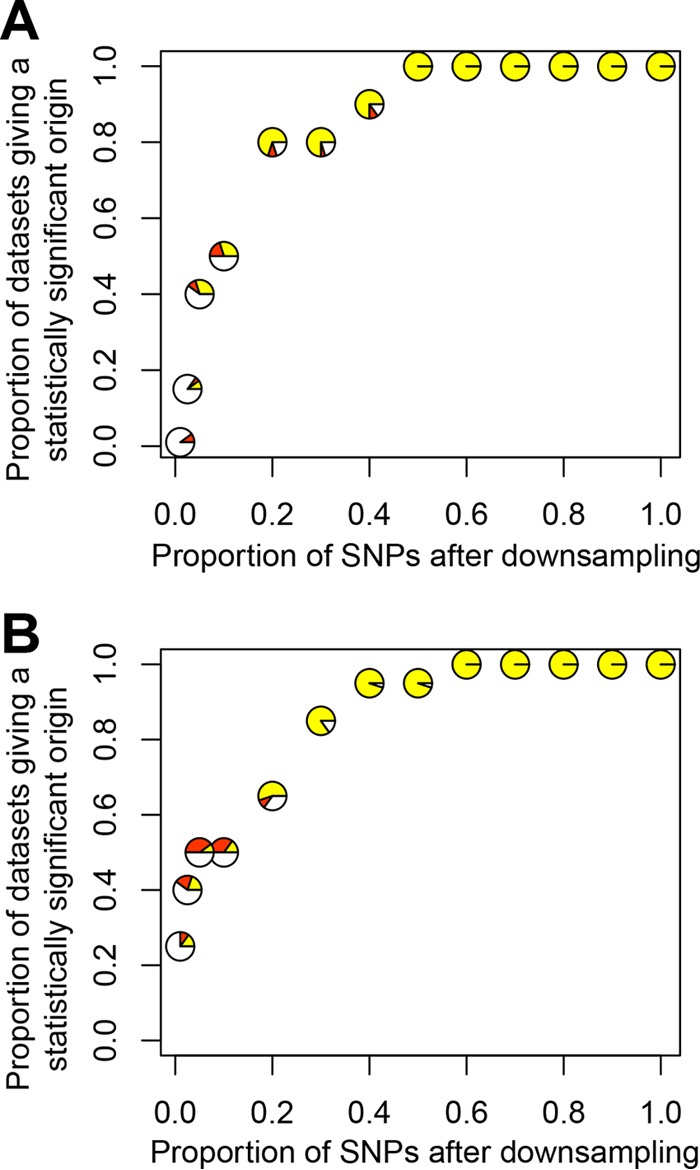
Power analysis of signatures of range expansion. USA300-NAE SNPs were randomly downsampled to make 20 new data sets for each bin of downsampled SNPs and tested for a significant origin by using the signature of a decrease in θ_π_ (panel A) or an increase in Ψ (panel B). Yellow represents those data sets giving a significant origin in the Pennsylvania region (i.e., Pennsylvania, Ohio, New York, or Massachusetts), red represents any other significant origin, and white represents a nonsignificant origin.

As a neutral demographic process, range expansion is expected to produce signatures across the genome. Although the two signatures of range expansion described above represent averages across SNPs, additional studies were done to confirm their genome-wide distribution. First, we separately studied the 1,413 nSNPs and the 516 sSNPs. These subsets of SNPs each gave an eastern origin of USA300-NAE, with Pennsylvania, Ohio, or Massachusetts as the origin, depending on the signature (Tables S7 and S8 in Text S1). Next, we randomly downsampled SNPs to gain insight into the minimum number of SNPs necessary to detect the eastern origin of USA300-NAE. Randomly downsampling to 1,300 and 780 SNPs for tests based on a decrease in θ_π_ and an increase in Ψ, respectively, gave an origin in the Pennsylvania region (i.e., Pennsylvania, Ohio, New York, or Massachusetts) at least 80% of the time, but the signatures rapidly decayed with further downsampling (Fig. 3). These findings verified that the signatures of the USA300-NAE range expansion were genome wide.

### Evidence for allele surfing of an FQ resistance allele.

Most derived alleles are expected to stay at a low frequency or go extinct during a range expansion because of the cumulative genetic drift caused by the serial founding of smaller populations along the expansion front (25). On rare occasions, a derived allele can persist through these serial founder events and reach a high frequency—this phenomenon is referred to as “allele surfing” (37). Alleles can surf regardless of their fitness effects, since strong genetic drift limits the efficacy of natural selection, but fitness can impact the long-term behavior of surfing alleles. For example, simulations indicate that in the colonized areas behind the expansion front, advantageous alleles tend to increase in frequency and can initiate a second expansion front from their own origin population (24, 38).

**FIG 4:**
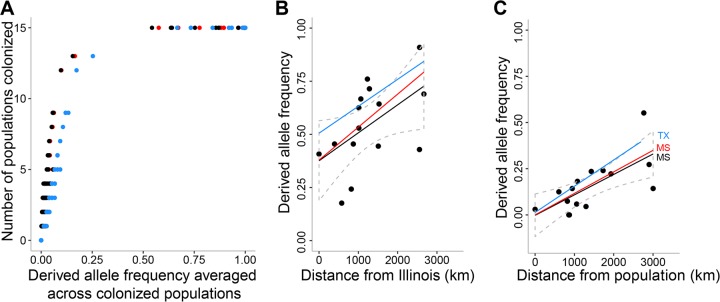
Identification of well-sampled derived alleles of USA300-NAE. Panel A shows the relationship between the frequency of derived alleles and the number of colonized populations when all isolates (from 2001 to 2011; black dots), recent isolates (from 2007 to 2011; red dots), or all isolates with allele frequencies binned into one of five 20% bins before averaging across populations (blue dots) were used. Panels B and C show the significant positive frequency gradients for the *gyrA* and *ssa-1* alleles, respectively. The data points, regression lines, and 95% confidence intervals are shown for all isolates (in black) along with the regression lines for recent isolates (red lines) and for all isolates with binned allele frequencies (blue lines). The different origin populations giving the best correlation with the different analyses are indicated in panel C.

To detect allele surfing, we focused our study on SNPs with well-sampled derived alleles. As found in previous simulations of range expansion (37), only a small fraction of derived alleles were well sampled (Fig. 4A, black dots); 18 of 2,595 (0.7%) derived alleles were sampled from 10 or more populations at frequencies averaging ≥10%. The same trend occurred when only recent isolates were considered (Fig. 4A, red dots) and when allele frequencies were binned to better control for unequal sample sizes among populations (Fig. 4A, blue dots). Frequency gradients were also rarely detected, as expected from simulations (37). Only 2 of these 18 well-sampled alleles had significant frequency gradients that were robust to the different sampling schemes and the false-discovery rate (FDR) (Table 3). These two alleles resulted from a nonsynonymous mutation in *gyrA* (Ser84Leu) with a positive gradient from Illinois (all isolates, Pearson *r* = 0.528, *P* = 0.02; recent isolates, Pearson *r* = 0.643, *P* = 0.0008; binned allele frequencies, Pearson *r* = 0.514, *P* = 0.018; all FDR-corrected *P* values with *n* = 15; Fig. 4B) and a synonymous mutation in *ssa-1*, which encodes staphylococcal secretory antigen 1, with a positive gradient from Mississippi or Texas, depending on the sampling scheme (all isolates, Pearson *r* = 0.690, *P* = 0.02; recent isolates, Pearson *r* = 0.720, *P* = 0.0075; binned allele frequencies, *r* = 0.643, *P* = 0.018; all FDR-corrected *P* values with *n* = 15; Fig. 4C). The *gyrA* allele was the only well-sampled allele to also have a significant positive gradient with distance from the Pennsylvania region (not shown). No correlation was detected between the *gyrA* allele frequency gradient and state-wide estimates of FQ use in humans (Table S9 in Text S1); thus, there is currently no evidence that the *gyrA* allele frequency gradient was induced by an environmental gradient.

**TABLE 3:** Unusual SNPs from various population genomic analyses

Category and TCH1516^a^ chromosome coordinate(s)	TCH1516 locus, gene	Comment
SNPs that are polymorphic in both USA300-NAE and USA300-SAE clades		
460	Intergenic before 0001	
14575	Intergenic 0008–0009	
233245	Coding 0210, hypothetical protein	nSNP
2542210	Intergenic 2401–2402	
SNPs with derived allele present in ≥10 populations with ≥10% average frequency and a significant frequency gradient		
7282	Coding 0006, *gyrA*	nSNP
2708710	Coding 2561, *ssa-1*	sSNP
SNPs within intervals that were recombined more often than expected under the CFML recombination model		
168833–169219	Intergenic 0159–0160	Recombined on 3 or 4 branches
672683–672727	Intergenic 0614–0615	Recombined on 3 or 4 branches
2600705	Intergenic 2461–2462	Recombined on 3 branches
2600727–2600792	Intergenic 2461–2462	Recombined on 3 or 4 branches
2680141–2680194	Intergenic 2536–2537	Recombined on 8 or 9 branches
2813570–2813582	Coding 2564, *sraP*	Recombined on 4 branches

a The TCH1516 locus designation has the prefix USA300HOU_.

It is important to note that the signatures of USA300-NAE range expansion do not depend on the *gyrA* allele (Table S10 in Text S1) and that the signatures predate the origin of the FQ-resistant subclade since a separate analysis with the isolates outside this clade also best supports an eastern origin (Table S11 in Text S1). In summary, a small number of successful alleles do not dominate the overall signature of an increased derived allele frequency; rather, the signature results from the more numerous mid- and low-frequency alleles that may expand geographically in more limited pulses. The *gyrA* allele may provide a rare example of an advantageous allele with two modes of spread during the USA300-NAE range expansion: one on the expansion front of USA300-NAE and the other centered on Illinois.

### Influence and patterns of recombination.

**FIG 5:**
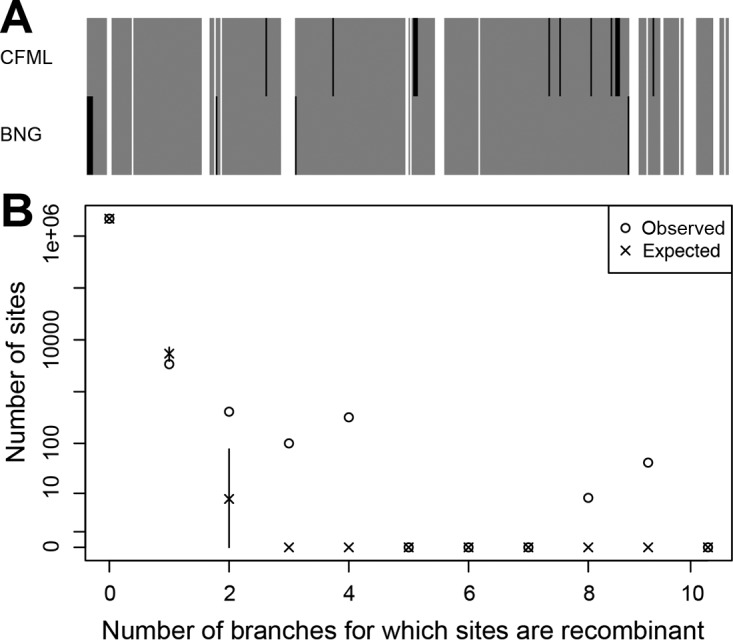
Recombination in USA300. Panel A shows 4,109 biallelic SNPs in columns and results of recombination analysis by CFML and BNG in rows. Gray columns indicate nonrecombinant SNPs according to both methods. White columns indicate recombinant SNPs according to both methods. Black columns indicate recombinant SNPs according to one method only. Panel B shows the distribution of the number of branches for which sites are recombinant as inferred by CFML (○) and as expected under the CFML model (×). In panel B, 95% confidence intervals are shown but the intervals are sometimes too small to be visible and sometimes the symbols for observed and expected results overlap.

The different model-based methods of ClonalFrameML (CFML) (39) and BratNextGen (BNG) (40) were used to detect recombination. In the full sample of 357 genomes, recombinant regions spanned 618 (15%) of 4,109 SNPs by CFML analysis, 574 (14%) of 4,109 SNPs by BNG analysis, and 493 (12%) of 4,109 SNPs by both methods (Fig. 5A). The inference of USA300-NAE’s origin described above was done after the removal of recombinant SNPs identified by CFML analysis (except for the average tip-to-tip distance that used recombination-corrected branch lengths). A similar result was obtained after the removal of recombinant SNPs identified by BNG analysis; in this case, Pennsylvania and the adjacent populations of Ohio and New York were the origins, depending on the signature (Table S12 in Text S1). Thus, the inferred eastern origin of USA300-NAE was robust to two different methods for detecting recombination. Of note, the inferred origin was decisively influenced by the presence of recombination. When all recombinant SNPs were included in the analysis, Florida was the origin with each signature (Table S13 in Text S1). Of the 10 branches on the ML phylogeny of USA300-NAE that were affected the most by recombination, 6 led to isolates exclusively from the southeastern United States. These results indicate that importation of divergent alleles into specific populations can, on average, increase their genetic diversity and decrease their derived allele frequency (possibly by displacing derived alleles with ancestral alleles). Thus, recombination detection is a crucial part of the analysis.

Two additional observations about recombination were made. First, some nucleotide sites were recombined more often than expected under the CFML model, especially those sites that had recombined in three or more branches of the phylogeny (Fig. 5B). Nearly all of these sites were intergenic, and transfer was not limited to USA300-NAE branches for two of these six loci (Table 3). The average tract length (standard deviation) of all recombinations was 83 (121) bp, which is shorter than that described at the species level for *S. aureus* (183 bp in reference 39 and 654 bp in reference 41). It is possible that the homoplasious distribution of some of these sites on the phylogeny has resulted from recurrent mutation rather than recurrent recombination, but their unusualness under the model indicates that they could be the result of positive selection. Second, the ratio of recombination to mutation events was 2.4 times as high in the FQ-resistant subclade of USA300-NAE as that in isolates outside this subclade that were largely FQ susceptible. The ρ/θ ratio (95% credibility interval) was 0.029 (0.022 to 0.38) for the FQ-resistant subclade and 0.012 (0.007 to 0.018) for the other isolates, despite similar population-scaled mutations rates (θ) of 0.00074 and 0.00077, respectively. Thus, diversification by recombination of core genome sequences has been higher in the FQ-resistant subclade than in the rest of USA300-NAE.

## DISCUSSION

Population genomics provides a complementary approach to phylogenomics when studying the origin and spread of pathogens. The serial founder model of range expansion is a population genetic model that has been used previously to infer an African origin of human populations (42, 43) and the affiliated microbial pathogens *Helicobacter pylori* and *Plasmodium falciparum* (44, 45). Through genome sequencing, we were able to extend this approach to study the much more recent origin and continental spread of the USA300-NAE clone.

Approximately half of the core nucleotide variation in USA300-NAE can be explained by geographic distance from Pennsylvania: 47 to 49% of the variation when considering all isolates and 54 to 63% of the variation when considering only recent isolates (Tables S5 and S6 in Text S1). Since it is the cumulative genetic drift caused by serial founding of smaller populations along the expansion front that is ultimately responsible for the signatures of range expansion (42), these correlations may provide an estimate of the impact of genetic drift in shaping USA300-NAE’s genetic variation. In general, the relative contribution of drift compared to other population processes that affect bacterial genetic variation is poorly understood outside certain contexts, such as obligate intracellular bacteria (46). Our results highlight a rarely considered but potentially common mechanism whereby drift may significantly impact bacterial genetic variation. All epidemic bacterial clones will expand their geographic range to some degree, but the signatures of range expansions may not always be detectable because of their dependence on demographic factors (28–30) that may differ among bacteria. Nonetheless, the drift that occurs during range expansion may have important long-term consequences for bacteria, such as an initially reduced capacity to respond to selection.

One limitation of our study was the lack of precise geographic coordinates of isolates within U.S. states that likely lowered the precision of the correlations noted above. In addition, although our study provides the most geographically diverse sample of USA300-NAE examined by genome sequencing to date, it is still a relatively sparse sample of U.S. populations. Even so, our results were sufficient to rule out potential confounders such as temporal differentiation and human population density as causes of the patterns observed.

While Pennsylvania was the most likely origin among our sampled populations in most analyses, the nearby populations of Ohio, New York, and Massachusetts were the most likely origins in certain subsets of analysis and ranked highly in most analyses. Considered together, our results strongly indicate that the origin of USA300-NAE is in the eastern and not the western United States. In fact, our results are incompatible with a West Coast origin of USA300-NAE. California, Oregon, and Washington populations had relatively high genetic differentiation, low genetic diversity, and a high derived allele frequency, which are expected at the edge of an expanding population and not at its origin. The result for the outlier population Hawaii was similar to that for the West Coast populations (Table S1 in Text S1). Therefore, our results do not validate anecdotes (47) or an analysis of the geographic center of publications on USA300 (48) that favor a shift from the West and Midwest toward the East. However, it is also important to point out that we have specifically characterized the origin and spread of the USA300-NAE clade, which has been the major cause of infections during this epidemic.

Rarely, a derived allele may propagate throughout a range expansion—this phenomenon has been called “allele surfing” (37) and has not been observed previously in natural populations of bacteria, to our knowledge. Hallatschek et al. (49) showed that the familiar process of sectoring of bacterial colonies on agar plates represents a laboratory version of allele surfing. Here, only two derived alleles individually had convincing positive frequency gradients consistent with some form of allele surfing. One of these alleles is from a silent mutation in *ssa-1* and is likely neutral, whereas the other is an FQ resistance allele in *gyrA* and likely confers a selective advantage. These findings also provide an explanation for a previous observation that FQ resistance may be less common in USA300 from Illinois than in USA300 from the East and West coasts (21)—because Illinois is nearer the origin of the FQ resistance allele and the higher frequencies were achieved as its own range expanded (Fig. 4B).

As a cautionary note, recombination of divergent alleles into some populations along an expansion front can introduce an artifact into the analysis. Thus, we focused on biallelic, nonrecombinant SNPs and demonstrated robustness of results to two different model-based methods for detecting recombination. For this same reason, we excluded accessory gene variation as a source of information for inferring origin. Our study used a broader geographic sample of USA300-NAE within the United States and detected more recombination than prior studies have (20, 21, 23), especially among isolates from the southeastern United States that have been poorly sampled in prior studies. In addition, we found that the FQ-resistant subclade of USA300-NAE had a higher recombination rate than the rest of USA300-NAE. The potential association between FQ resistance and recombination might be explained by molecular genetic and/or population genetic mechanisms. For example, FQs may stimulate recombination pathways in *S. aureus*, as observed in *Escherichia coli* (64), and the FQ-resistant strains would have a better ability to survive exposure to the antibiotic. Alternatively, import of divergent alleles along an expansion front may reflect the reduced efficacy of selection in serially founded populations, as observed in simulations and empirical studies of range expansion (24), and is more evident in the slightly more recently founded FQ-resistant subclade than in the rest of USA300-NAE.

In summary, our results narrow the geographic region of the origin of USA300-NAE to the eastern United States, specifically the Pennsylvania region, and identify several unusual polymorphisms that have persisted through the sequential bottlenecking of this clone’s spread across the United States. New results extend previous observations (20, 21) that underscore the importance of FQ resistance in the evolution of USA300-NAE. Finally, our study suggests that the genetic drift attributed to geographic range expansion can be a potent evolutionary force in bacteria.

## MATERIALS AND METHODS

### Bacterial isolates.

A total of 357 isolates from 22 U.S. states and territories and seven other countries (Argentina, Australia, Columbia, Denmark, Ecuador, Germany, and Ireland) were included in this study (Data Set S1). All isolates were determined to be USA300 or closely related by screening (or rescreening) for the *spa* type and the presence of *mecA* and PVL-associated genes by previously described methods (50). Ciprofloxacin susceptibility testing was done by standard disk diffusion methods (65). Over half of the isolates, 191 (54%) of 357, were from two surveys of MRSA diversity in the United States (51); these isolates were collected from 81 nasal and 110 blood specimens from unique patients in 15 states in 2009 to 2011. The remaining isolates were selected to increase sample sizes or to provide a sample for seven states and Puerto Rico (103 isolates), were early reference isolates from the United States (26 isolates), or were international isolates (36 isolates). In addition, the reference genome sequence of strain TCH1516 (52) was included. The genomes of 322 isolates were newly sequenced, and those of 35 isolates were from a prior study (16).

### Genome sequencing, alignment, and assembly.

Sequencing was performed with Illumina (San Diego, CA) MiSeq instruments. Genomic DNA was isolated with Qiagen DNeasy Blood and Tissue kits. Sequencing libraries were prepared with Illumina Nextera XT DNA sample preparation kits (250-bp paired-end reads). MiSeq Reporter was used to demultiplex and trim adapters from the sequence reads. CLC Genomics Workbench v7 (Qiagen, Aarhus, Denmark) was used to filter reads for minimum quality (base quality, ≥Q13; number of ambiguities, ≤2; read length, ≥15 bp), align reads with the reference genome of strain TCH1516, exclude ambiguously aligned and duplicate reads, and assemble genomes *de novo*. Read and assembly information for each genome is presented in Data Set S1.

### Variant calling, filtering, and functional assignment.

Aligned reads were coordinate sorted, realigned around insertion-deletion polymorphisms (indels), and compressed in size with Picard v1.85 and GATK v2.3.9 (53). Repetitive regions of ≥50 bp with ≥80% nucleotide identity, identified through a pairwise megablast search of the reference genome against itself, and five known mobile genetic elements were excluded from variant calling. Multiallelic and biallelic SNPs and indels were called with GATK. Variants with a base quality of ≥Q30, a read depth of ≥3, and a base “homozygosity” of ≥75% in all genomes were retained. SnpEff v4 (54) was used with the annotation of the reference genome to assign functional effects to the variants. The invariant core was filtered to the same stringency as the variants and was merged with the filtered biallelic SNPs to produce a multi-fasta alignment that was used for phylogenomic analysis and recombination detection.

### Phylogenomic analysis and recombination detection.

An ML phylogeny was generated with PhyML v3.0 (55) under an HKY + Γ substitution model. Subtree pruning and a regrafting search of the tree space were performed with a BioNJ starting tree and 10 random starting trees. Node support was evaluated by using 100 bootstrap replicates. To detect recombinant sequences, estimate recombination parameters, and correct branch lengths, CFML (39) was used with the ML phylogeny and default parameters under its standard model. BNG (40) was used to detect recombinations with a run of 20 iterations, and the significance of the recombinant segments was determined with 100 permutations and a cutoff *P* value of 0.05.

A time-stamped Bayesian phylogeny was generated with BEAST v1.7.5 (56) under an HKY + Γ substitution model and an uncorrelated lognormal relaxed molecular clock. For these analyses, nonrecombinant sequences were used. Two demographic models were considered, a constant-size coalescent model and a Bayesian skyline model. For each model, Markov chain Monte Carlo chains were run four times, each run for 200 million steps with sampling every 20,000 steps. Convergence and mixing were checked with Tracer v1.5. Runs were combined after the removal of 10% of the samples as a burn-in with LogCombiner v1.7.5. The Bayesian skyline model was a better fit (Bayes factor, 4.82) and was therefore used for this study. A maximum clade credibility tree was generated with TreeAnnotator v1.7.5.

### Population genomic analysis.

Genetic differentiation between populations was measured with Hudson’s F_ST_ (57) across biallelic, nonrecombinant SNPs and the significance of pairwise F_ST_ values was tested with 10,000 permutations and a Bonferroni *P* value. Principal-component analysis of the pairwise F_ST_ matrix was done with the princomp function of the built-in R stats package (58). Geographic distance between populations was measured by using the haversine great-circle distance (in kilometers) between the centers of U.S. states and territories with the R package geosphere (58). The numbers of airline passengers and migrants between U.S. states were obtained from the U.S. Bureau of Transportation Statistics (https://www.transtats.bts.gov/Fields.asp?Table_ID=310) and the U.S. Census Bureau (https://www.census.gov/data/tables/time-series/demo/geographic-mobility/state-to-state-migration.html), respectively. The comparisons of all isolates (2001 to 2011 isolation dates) were done by using 2001 to 2011 passenger data and 2005 to 2011 (earliest available) migrant data, whereas the comparisons of recent isolates (2007 to 2011 isolation dates) used 2007 to 2011 passenger and migrant data. The numbers of passengers or migrants, respectively, between any two U.S. states are asymmetrical and can differ by orders of magnitude but are significantly correlated (passengers, Pearson *r* = 0.999, *P* = 2.2e-16; migrants, Pearson *r* = 0.843, *P* = 2.2e-16; two-tailed test with *n* = 105 pairs), so (log) totals were used. Mantel and partial Mantel tests with 10,000 permutations were performed with the R package ecodist (59).

Given the evidence for nonequilibrium populations, we first validated appropriate measurements of genetic diversity to study range expansion. Watterson’s estimator (θ_w_), which is more sensitive to recent mutations (60), was correlated with sample size (Pearson *r* = 0.707, *P* = 0.003, two-tailed test with *n* = 15) and was therefore inappropriate for our study since the diversity would reflect sample size rather than distance from the origin. Average pairwise difference (θ_π_), which is more sensitive to older mutations (60), was uncorrelated with sample size (Pearson *r* = −0.135, *P* = 0.63, two-tailed test with *n* = 15) and was therefore used in our study. The average tip-to-tip (patristic) distance on the ML tree, which is analogous to θ_π_, was also uncorrelated with sample size (Pearson *r* = 0.119, *P* = 0.67, two-tailed test with *n* = 15) and was used in our study. Nucleotide diversity and Tajima’s D were calculated with the R package pegas (61), whereas tip-to-tip distance was calculated with the R packages ape (62) and phangorn (63). The origin was inferred by linear regression as the population with the most negative correlation between genetic diversity and geographic distance from the origin (42, 44) with the built-in R stats package (58). A separate analysis to estimate P_perm_ was obtained by randomly shuffling isolates between populations and determining the best origin 1,000 times. For graphic display of results, correlations between sampling points were interpolated by kriging with ArcGIS v10.2.2 (ESRI, Redlands, CA) with the Spatial Analyst Toolbox and a default grid size of 0.78.

To study derived allele frequency, we first polarized the ancestry of alleles in USA300-NAE by using the USA300-SAE sister clade as an outgroup and assigning the allele common to both clades as ancestral and the allele unique to USA300-NAE as derived. Four biallelic, nonrecombinant SNPs were polymorphic in both clades and thus were excluded (Table 3). The directionality index (Ψ) was then calculated by using the R source code modified from the rangeExpansion package (27). Ψ is a pairwise measurement of the difference in average derived allele frequency between populations i and j. Ψ is negative when population i is closer to the origin and positive when population j is closer to the origin. Ψ is zero when the populations are the same distance from the origin and when there is no range expansion. For each pair of populations, only derived alleles present in both populations were used and sample size differences were handled by repeatedly downsampling to the smallest sample size in the pair. The origin was inferred by nonlinear regression as the population with the most positive correlation between Ψ and the distance from the origin (27). In addition, we report the origin inferred from a linear regression between Ψ and the distance from the origin, along with P_perm_ from the 1,000 data sets where isolates were randomly shuffled between populations.

To test the relationship between human population density and signatures of range expansion, we used population density data for the years 2000 and 2010 from the U.S. Census Bureau (https://www.census.gov/2010census/data/apportionment-dens-text.php). A multivariate regression analysis of nucleotide diversity with distance from Pennsylvania was performed with the built-in R stats package (58) with population density as a covariate. The *P* values reported in population genomic analyses were one tailed, unless otherwise noted, because specific positive or negative relationships were being tested. For the finer-scale study of derived allele frequency, the FDR was controlled with the Benjamini-Hochberg correction of *P* values with the p.adjust function of the built-in R stats package (58).

### Accession number(s).

The sequence reads obtained in this study were submitted to the NCBI Sequence Read Archive (BioProject PRJNA330544).
