# Supplementary material for: Range Expansion and the Origin of USA300 North American Epidemic Methicillin-Resistant Staphylococcus aureus
Source: mBio. 2018 Jan 2;9(1):e02016-17. doi: 10.1128/mBio.02016-17 (PMC5750399; doi:10.1128/mBio.02016-17)
Supplement: TEXT S1 [file mbo001183654s1.pdf]

## SUPPLEMENTAL TEXT

**Phylogeographic analysis.** The ML phylogeny with branch lengths corrected for recombination was used for ancestral state reconstruction of geographic location of USA300-NAE under parsimony and likelihood. Calculations were done with Mesquite v2.75 (1) and MacClade v4.08 (Sinauer Associates, Sunderland, MA). The two most basal isolates of USA300-NAE in our sample were from Indiana and Mississippi (Fig. 1 circle 3). Mississippi was the most abundant sample of USA300-NAE at 33 isolates and was reconstructed as its origin under parsimony and with 55% likelihood. However, in 100 subsamples where the Mississippi sample was rarefied to 16 isolates (the average of 18 USA300-NAE populations with ≥6 isolates each), Mississippi was the unambiguous origin only 42% of the time under parsimony and with an average of 26% likelihood. Mississippi was especially oversampled among early isolates, accounting for 16 of 36 (44%) isolates collected between 2001-2005, but only 17 of 229 (7.4%) isolates collected between 2007-2011. When only the recent isolates from 2007-2011 were considered, Mississippi was not reconstructed as the origin under parsimony or likelihood. Despite the phylogenomic uncertainty in origin, the distribution of US states and territories on the ML phylogeny was non-random (Slatkin-Maddison test, $P<0.001$). This signal was likely the result of "microclustering" where two or more isolates from the same US state or territory were adjacent on the phylogeny (Fig. 1 circle 3), as 443 of 515 (86%) unambiguous changes (migrations) and stasis events under parsimony were within US states and territories.

The clade distribution of the international isolates included 20 isolates of USA300-NAE (from Australia, Ireland, Germany and Denmark), all nine isolates of USA300-SAE (from Colombia and Ecuador), one Ecuador isolate of early-branching USA300, and six non-USA300 isolates (from Argentina and Denmark). Among the 20 isolates of USA300-NAE, the three isolates from Germany formed a microcluster, as did two isolates from Ireland, but the 15 remaining isolates were in separate microclusters with US isolates, consistent with prior conclusions of multiple exports of USA300 from the US to the eastern hemisphere (2,3).

**Genetic and geographic outliers.** Using 2599 biallelic, nonrecombinant SNPs, the average (SD) pairwise Hudson's $F_{ST}$ between the 18 populations with sample size ≥6 isolates was 0.0583 (0.0472). Montana was identified as a genetic outlier population, as its average pairwise $F_{ST}$ was

2.6x higher and it was clearly distinguished in a principal components analysis (Fig. S2). Further sample information was unavailable to assess whether the Montana isolates were from a remote host population(s), but their genetic information indicates that the differentiation was not due to unique recombination events. Puerto Rico was the second most differentiated population (Fig. S2). Hawaii and Puerto Rico were considered to be geographic outliers due to the large bodies of water separating these populations from the nearest continental US populations. For these reasons, the Montana, Hawaii and Puerto Rico populations were excluded from subsequent analysis.

Nonetheless, inclusion of the Montana, Hawaii and Puerto Rico outlier populations did not qualitatively alter our conclusion of an eastern US origin of USA300-NAE though statistical significance was affected. The isolation by distance was weaker when these three populations were included (Mantel test, r= 0.265, P= 0.106, n=153 pairs). When these three populations were included in the test for range expansion and inference of origin, New York was the most likely origin based on decrease in $\theta_\pi$ (Pearson r=-0.510, P=0.015, $P_{perm}$=0.14, n=18), decrease in tip-to-tip distance (Pearson r=-0.516, P=0.014, $P_{perm}$=0.15, n=18), and increase in $\Psi$ with linear regression (Pearson r=0.505, P=0.016, $P_{perm}$=0.17, n=18) (Table S1). The nonlinear regression on $\Psi$ gave the unsampled population of New Hampshire as the best origin (Pearson r=0.535, $P_{perm}$=0.15, n=18) (Table S1).

References for Supplemental text


1. Maddison WP & Maddison DR (2016) Mesquite: a modular system for evolutionary analysis. Version 3.10 http://mesquiteproject.org
2. Von Dach E*, et al.* (2016) Comparative genomics of community-associated methicillin-resistant *Staphylococcus aureus* shows the emergence of clone ST8-USA300 in Geneva, Switzerland. *J Infect Dis* 213(9):1370-1379.
3. Glaser P*, et al.* (2016) Demography and intercontinental spread of the USA300 community-acquired methicillin-resistant *Staphylococcus aureus* lineage. *MBio* 7(1):e02183-02115.

Figure S1. Time-stamped Bayesian phylogeny of USA300 inferred using BEAST with non-recombinant genome sequences. Branch color indicates posterior probability of tip-ward node. Bars on nodes indicate 95% credibility intervals for node ages. Three colored bars adjacent to the tree indicate the *S. aureus* clade, fluoroquinolone resistance mutation and the geographic source of isolation (see color index).

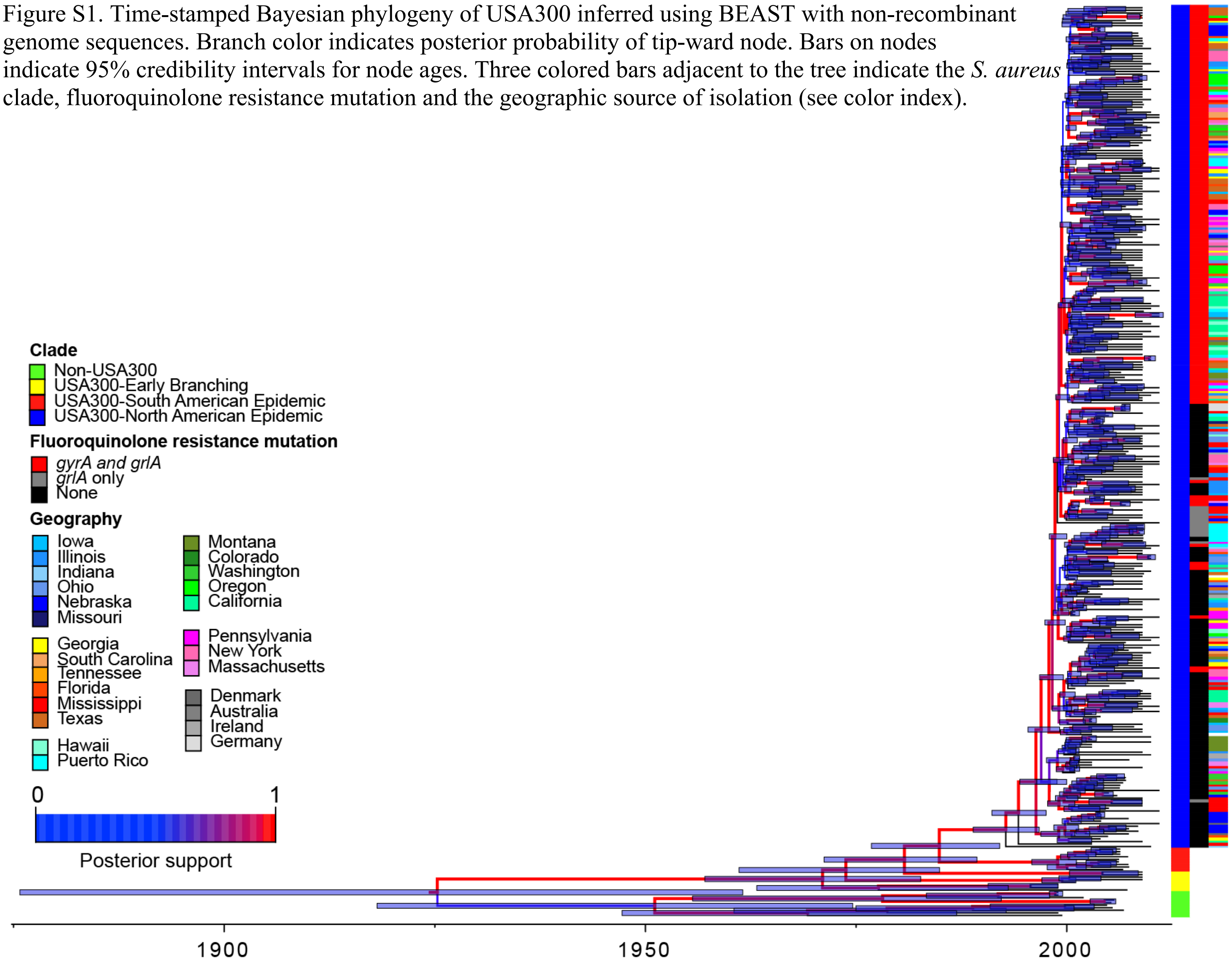

Figure S2. Principal components analysis based on the pairwise Hudson's $F_{ST}$ between 18 populations of USA300- NAE.

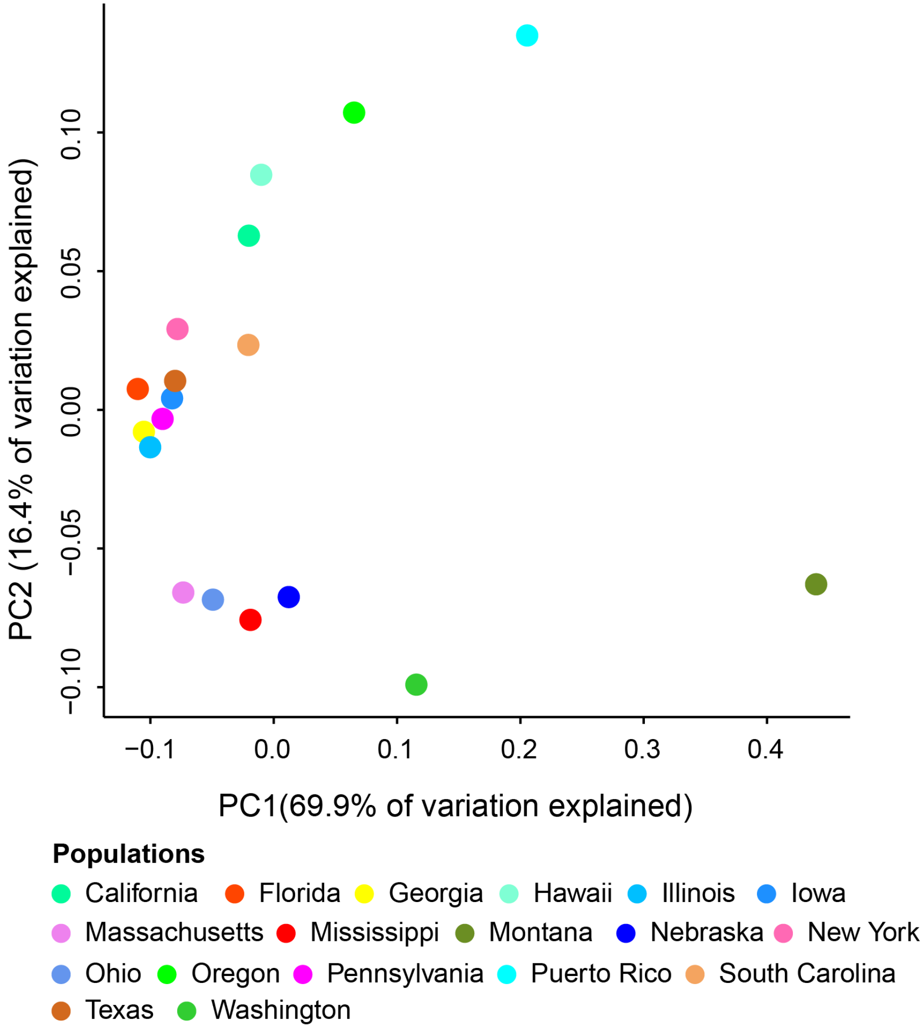

Table S1. Signatures of range expansion and origin of USA300-NAE with 18 population dataset.

| **Decrease in genetic diversity ($\theta_\pi$)** | | | | **Decrease in genetic diversity (tip-to-tip distance)** | | | | **Increase in derived allele frequency ($\Psi$)*** | | | |
|---|---|---|---|---|---|---|---|---|---|---|---|
| **Origin population** | **r** | **P** | **$P_{perm}$** | **Origin population** | **r** | **P** | **$P_{perm}$** | **Origin population** | **r** | **P** | **$P_{perm}$** |
| NY | -0.510 | 0.0154 | 0.142 | NY | -0.516 | 0.0142 | 0.148 | NY | 0.505 | 0.0162 | 0.165 |
| MA | -0.506 | 0.0160 | | MA | -0.509 | 0.0155 | | MA | 0.505 | 0.0163 | |
| PA | -0.501 | 0.0171 | | PA | -0.507 | 0.0159 | | PA | 0.498 | 0.0178 | |
| OH | -0.491 | 0.0193 | | OH | -0.499 | 0.0175 | | OH | 0.481 | 0.0218 | |
| SC | -0.413 | 0.0443 | | IL | -0.430 | 0.0373 | | SC | 0.418 | 0.0421 | |
| IL | -0.404 | 0.0482 | | SC | -0.415 | 0.0436 | | GA | 0.382 | 0.0589 | |
| GA | -0.383 | 0.0583 | | IA | -0.402 | 0.0490 | | IL | 0.362 | 0.0700 | |
| IA | -0.371 | 0.0648 | | GA | -0.394 | 0.0528 | | PR | 0.336 | 0.0867 | |
| FL | -0.326 | 0.0935 | | NE | -0.345 | 0.0807 | | FL | 0.335 | 0.0873 | |
| MS | -0.307 | 0.1080 | | MS | -0.343 | 0.0820 | | IA | 0.304 | 0.1102 | |
| NE | -0.299 | 0.1137 | | FL | -0.337 | 0.0856 | | MS | 0.280 | 0.1305 | |
| PR | -0.294 | 0.1178 | | PR | -0.318 | 0.0995 | | NE | 0.214 | 0.1972 | |
| MT | -0.210 | 0.2019 | | TX | -0.248 | 0.1604 | | TX | 0.155 | 0.2701 | |
| TX | -0.198 | 0.2161 | | MT | -0.231 | 0.1782 | | MT | 0.107 | 0.3361 | |
| WA | 0.036 | 0.5562 | | WA | 0.034 | 0.5535 | | OR | -0.174 | 0.7549 | |
| OR | 0.072 | 0.6119 | | OR | 0.063 | 0.5986 | | WA | -0.194 | 0.7795 | |
| CA | 0.135 | 0.7028 | | CA | 0.100 | 0.6536 | | CA | -0.216 | 0.8053 | |
| HI | 0.381 | 0.9407 | | HI | 0.395 | 0.9477 | | HI | -0.404 | 0.9519 | |

*NH (latitude: 43.8836, longitude: -71.9776) was best with nonlinear regression of $\Psi$ (r=0.535, $P_{perm}$=0.154)

Table S2. Population genomic summary statistics for 15 populations of USA300-NAE (recent isolates).

| **Population** | **No. isolates** | **Average pairwise $F_{ST}$** | **$\theta_\pi$** | **$\theta_W$** | **Tajima's D** | **P (D=0)** | **Average tip-to-tip distance ($x10^{-5}$)** | **Sum pairwise Ψ** |
|---|---|---|---|---|---|---|---|---|
| All 15 | 229 | 0.0321 | 0.0118 | 0.1440 | -2.9377 | 0.0033 | 1.2678 | - |
| IL | 27 | 0.0190 | 0.0104 | 0.0331 | -2.7086 | 0.0068 | 1.2196 | 0.4737 |
| NY | 25 | 0.0254 | 0.0119 | 0.0311 | -2.4655 | 0.0137 | 1.3919 | -0.8111 |
| CA | 23 | 0.0465 | 0.0095 | 0.0219 | -2.2983 | 0.0215 | 1.0973 | 1.0905 |
| TX | 19 | 0.0276 | 0.0105 | 0.0239 | -2.3636 | 0.0181 | 1.2252 | 0.2931 |
| MS | 17 | 0.0253 | 0.0112 | 0.0231 | -2.2151 | 0.0268 | 1.2836 | -0.0732 |
| OH | 17 | 0.0311 | 0.0151 | 0.0319 | -2.2684 | 0.0233 | 1.7133 | -2.0332 |
| GA | 16 | 0.0187 | 0.0134 | 0.0278 | -2.2684 | 0.0233 | 1.5136 | -1.3311 |
| PA | 15 | 0.0224 | 0.0137 | 0.0262 | -2.1131 | 0.0346 | 1.5356 | -1.5834 |
| NE | 14 | 0.0566 | 0.0121 | 0.0194 | -1.6914 | 0.0908 | 1.3468 | 0.1218 |
| FL | 14 | 0.0175 | 0.0096 | 0.0201 | -2.3475 | 0.0189 | 1.0774 | 1.1612 |
| IA | 11 | 0.0256 | 0.0099 | 0.0156 | -1.7511 | 0.0799 | 1.0925 | 0.9153 |
| OR | 11 | 0.0609 | 0.0095 | 0.0138 | -1.5072 | 0.1318 | 1.0406 | 0.7772 |
| MA | 9 | 0.0248 | 0.0131 | 0.0183 | -1.4688 | 0.1419 | 1.4067 | -0.8583 |
| SC | 6 | 0.0381 | 0.0116 | 0.0133 | -0.8322 | 0.4053 | 1.1667 | -0.4173 |
| WA | 5 | 0.0417 | 0.0093 | 0.0096 | -0.2299 | 0.8182 | 0.9058 | 2.2748 |

Average pairwise $F_{ST}$, $\theta_\pi$, $\theta_W$ and D was based on 2599 biallelic, non-recombinant SNPs.
Average tip-to-tip distance reflects branch lengths on a ML tree that has been corrected for recombinant sites.
Sum of pairwise Ψ was based on 2595 biallelic, non-recombinant SNPs where ancestral and derived alleles were assigned.

Table S3. Pairwise Hudson's $F_{ST}$ between 15 populations of USA300-NAE using all isolates (lower triangle) and recent isolates (upper triangle).

| | **CA** | **FL** | **GA** | **IA** | **IL** | **MA** | **MS** | **NE** | **NY** | **OH** | **OR** | **PA** | **SC** | **TX** | **WA** |
|---|---|---|---|---|---|---|---|---|---|---|---|---|---|---|---|
| **CA** | 0.000 | 0.029 | 0.032 | 0.027 | 0.039 | 0.038 | 0.049 | 0.093 | 0.040 | 0.058 | 0.070 | 0.037 | 0.064 | 0.046 | 0.075 |
| **FL** | 0.026 | 0.000 | 0.007 | 0.008 | 0.001 | 0.010 | 0.007 | 0.053 | 0.006 | 0.024 | 0.036 | 0.009 | 0.030 | 0.006 | 0.036 |
| **GA** | 0.030 | 0.007 | 0.000 | 0.011 | 0.002 | 0.016 | 0.015 | 0.043 | 0.015 | 0.022 | 0.055 | -0.007 | 0.019 | 0.014 | 0.037 |
| **IA** | 0.025 | 0.008 | 0.011 | 0.000 | 0.004 | 0.018 | 0.019 | 0.059 | 0.022 | 0.022 | 0.068 | 0.018 | 0.038 | 0.023 | 0.047 |
| **IL** | 0.036 | 0.001 | 0.002 | 0.004 | 0.000 | 0.014 | 0.005 | 0.049 | 0.012 | 0.016 | 0.057 | 0.010 | 0.026 | 0.011 | 0.039 |
| **MA** | 0.034 | 0.010 | 0.016 | 0.018 | 0.014 | 0.000 | 0.011 | 0.037 | 0.020 | 0.010 | 0.073 | 0.020 | 0.045 | 0.032 | 0.028 |
| **MS** | 0.070 | 0.028 | 0.026 | 0.032 | 0.020 | 0.006 | 0.000 | 0.053 | 0.015 | 0.022 | 0.061 | 0.021 | 0.037 | 0.018 | 0.046 |
| **NE** | 0.074 | 0.036 | 0.032 | 0.046 | 0.036 | 0.026 | 0.023 | 0.000 | 0.068 | 0.033 | 0.116 | 0.053 | 0.078 | 0.068 | 0.046 |
| **NY** | 0.038 | 0.006 | 0.015 | 0.022 | 0.012 | 0.020 | 0.045 | 0.053 | 0.000 | 0.038 | 0.050 | 0.016 | 0.011 | 0.016 | 0.052 |
| **OH** | 0.054 | 0.024 | 0.022 | 0.022 | 0.016 | 0.010 | 0.010 | 0.027 | 0.038 | 0.000 | 0.081 | 0.022 | 0.046 | 0.040 | 0.033 |
| **OR** | 0.071 | 0.036 | 0.055 | 0.068 | 0.057 | 0.073 | 0.102 | 0.099 | 0.050 | 0.081 | 0.000 | 0.055 | 0.072 | 0.045 | 0.074 |
| **PA** | 0.039 | 0.010 | -0.005 | 0.017 | 0.009 | 0.020 | 0.030 | 0.041 | 0.019 | 0.019 | 0.061 | 0.000 | 0.031 | 0.022 | 0.029 |
| **SC** | 0.063 | 0.030 | 0.019 | 0.038 | 0.026 | 0.045 | 0.059 | 0.065 | 0.011 | 0.046 | 0.072 | 0.032 | 0.000 | 0.030 | 0.043 |
| **TX** | 0.041 | 0.003 | 0.010 | 0.018 | 0.006 | 0.026 | 0.037 | 0.046 | 0.015 | 0.031 | 0.047 | 0.019 | 0.027 | 0.000 | 0.041 |
| **WA** | 0.107 | 0.070 | 0.060 | 0.075 | 0.068 | 0.039 | 0.038 | 0.043 | 0.088 | 0.037 | 0.126 | 0.058 | 0.082 | 0.073 | 0.000 |

Pairwise $F_{ST}$ was evaluated with 10,000 permutations. Comparisons that are significant by Bonferroni P-value are highlighted in yellow.

Table S4. Relationships between genetic differentiation (Weir and Cockerham's $F_{ST}$), geographic distance, and connectivity from all pairwise comparisons of 15 populations of USA300-NAE.

| | | **All isolates (2001-2011)** | | **Recent isolates (2007-2011)** | |
|---|---|---|---|---|---|
| **Test** | **Predictor of genetic distance** | **r** | **P** | **r** | **P** |
| Mantel | Geographic distance | 0.364 | 0.0097 | 0.288 | 0.066 |
| | Log total airline passengers | -0.379 | 0.034 | -0.345 | 0.059 |
| | Log total migrants | -0.237 | 0.113 | -0.204 | 0.161 |
| Partial Mantel | Geographic distance accounting for log total airline passengers | 0.334 | 0.025 | 0.252 | 0.146 |
| | Log total airline passengers accounting for geographic distance | -0.351 | 0.056 | -0.318 | 0.080 |

Mantel and partial Mantel tests were evaluated with 10,000 permutations.

Table S5. Signatures of range expansion and origin of USA300-NAE with 15 population dataset (all isolates).

| **Decrease in genetic diversity ($\theta_\pi$)** | | | | **Decrease in genetic diversity (tip-to-tip distance)** | | | | **Increase in derived allele frequency ($\Psi$)*** | | | |
|---|---|---|---|---|---|---|---|---|---|---|---|
| **Origin population** | **r** | **P** | **$P_{perm}$** | **Origin population** | **r** | **P** | **$P_{perm}$** | **Origin population** | **r** | **P** | **$P_{perm}$** |
| PA | -0.697 | 0.0019 | 0.024 | PA | -0.684 | 0.0025 | 0.031 | PA | 0.667 | 0.0033 | 0.029 |
| MA | -0.696 | 0.0020 | | NY | -0.683 | 0.0025 | | NY | 0.664 | 0.0035 | |
| NY | -0.695 | 0.0020 | | OH | -0.677 | 0.0028 | | MA | 0.663 | 0.0035 | |
| OH | -0.684 | 0.0025 | | MA | -0.673 | 0.0030 | | OH | 0.650 | 0.0043 | |
| SC | -0.558 | 0.0153 | | SC | -0.527 | 0.0218 | | SC | 0.543 | 0.0182 | |
| GA | -0.490 | 0.0320 | | GA | -0.479 | 0.0355 | | GA | 0.471 | 0.0382 | |
| IL | -0.432 | 0.0541 | | IL | -0.475 | 0.0367 | | IL | 0.396 | 0.0718 | |
| FL | -0.403 | 0.0680 | | FL | -0.384 | 0.0786 | | FL | 0.395 | 0.0725 | |
| MS | -0.262 | 0.1727 | | MS | -0.315 | 0.1265 | | MS | 0.234 | 0.2008 | |
| IA | -0.240 | 0.1950 | | IA | -0.306 | 0.1338 | | IA | 0.200 | 0.2374 | |
| NE | 0.099 | 0.6370 | | NE | -0.013 | 0.4811 | | TX | -0.106 | 0.6461 | |
| TX | 0.126 | 0.6731 | | TX | 0.029 | 0.5406 | | NE | -0.140 | 0.6912 | |
| WA | 0.570 | 0.9868 | | CA | 0.523 | 0.9772 | | CA | -0.546 | 0.9824 | |
| OR | 0.578 | 0.9879 | | OR | 0.555 | 0.9841 | | OR | -0.561 | 0.9852 | |
| CA | 0.589 | 0.9895 | | WA | 0.560 | 0.9850 | | WA | -0.593 | 0.9901 | |

*NY (latitude: 41.6967, longitude: -73.5591) was best with nonlinear regression of $\Psi$ (r=0. 686, $P_{perm}$=0.029)

Table S6. Signatures of range expansion and origin of USA300-NAE with 15 population dataset (recent isolates).

| **Decrease in genetic diversity ($\theta_\pi$)** | | | | **Decrease in genetic diversity (tip-to-tip distance)** | | | | **Increase in derived allele frequency ($\Psi$)*** | | | |
|---|---|---|---|---|---|---|---|---|---|---|---|
| **Origin population** | **r** | **P** | **$P_{perm}$** | **Origin population** | **r** | **P** | **$P_{perm}$** | **Origin population** | **r** | **P** | **$P_{perm}$** |
| PA | -0.748 | 0.0007 | 0.008 | PA | -0.732 | 0.0008 | 0.010 | PA | 0.786 | 0.0003 | 0.006 |
| OH | -0.731 | 0.0010 | | OH | -0.719 | 0.0008 | | OH | 0.766 | 0.0004 | |
| NY | -0.730 | 0.0010 | | NY | -0.719 | 0.0013 | | NY | 0.762 | 0.0005 | |
| MA | -0.727 | 0.0011 | | MA | -0.706 | 0.0017 | | MA | 0.759 | 0.0005 | |
| SC | -0.596 | 0.0095 | | SC | -0.568 | 0.0106 | | SC | 0.662 | 0.0036 | |
| GA | -0.536 | 0.0196 | | GA | -0.526 | 0.0164 | | GA | 0.600 | 0.0090 | |
| IL | -0.469 | 0.0390 | | IL | -0.506 | 0.0230 | | FL | 0.503 | 0.0279 | |
| FL | -0.435 | 0.0527 | | FL | -0.423 | 0.0434 | | IL | 0.492 | 0.0313 | |
| MS | -0.363 | 0.0916 | | MS | -0.400 | 0.0549 | | MS | 0.434 | 0.0533 | |
| IA | -0.258 | 0.1767 | | IA | -0.312 | 0.1271 | | IA | 0.235 | 0.1993 | |
| NE | 0.087 | 0.6214 | | NE | 0.008 | 0.4614 | | TX | -0.032 | 0.5457 | |
| TX | 0.095 | 0.6321 | | TX | 0.020 | 0.5303 | | NE | -0.161 | 0.7164 | |
| WA | 0.577 | 0.9879 | | CA | 0.565 | 0.9887 | | CA | -0.652 | 0.9958 | |
| OR | 0.603 | 0.9913 | | WA | 0.583 | 0.9909 | | OR | -0.656 | 0.9961 | |
| CA | 0.628 | 0.9939 | | OR | 0.588 | 0.9912 | | WA | -0.687 | 0.9977 | |

*PA (latitude: 41.2498, longitude: -76.9675) was best with nonlinear regression of $\Psi$ (r=0.795, $P_{perm}$=0.005)

Table S7. Signatures of range expansion and origin of USA300-NAE with 15 population dataset (non-synonymous SNPs).

| Decrease in genetic diversity ($\theta_\pi$) | | | | Increase in derived allele frequency ($\Psi$)* | | | |
|---|---|---|---|---|---|---|---|
| **Origin population** | **r** | **P** | **$P_{perm}$** | **Origin population** | **r** | **P** | **$P_{perm}$** |
| MA | -0.694 | 0.0020 | 0.018 | MA | 0.544 | 0.0180 | 0.097 |
| PA | -0.688 | 0.0023 | | NY | 0.537 | 0.0194 | |
| NY | -0.687 | 0.0023 | | PA | 0.530 | 0.0212 | |
| OH | -0.666 | 0.0034 | | OH | 0.471 | 0.0381 | |
| SC | -0.556 | 0.0158 | | SC | 0.442 | 0.0493 | |
| GA | -0.492 | 0.0312 | | GA | 0.391 | 0.0750 | |
| FL | -0.411 | 0.0641 | | IL | 0.337 | 0.1098 | |
| IL | -0.409 | 0.0650 | | FL | 0.334 | 0.1118 | |
| MS | -0.266 | 0.1687 | | IA | 0.245 | 0.1892 | |
| IA | -0.220 | 0.2151 | | MS | 0.212 | 0.2240 | |
| TX | 0.094 | 0.6305 | | NE | 0.091 | 0.3741 | |
| NE | 0.115 | 0.6588 | | TX | 0.022 | 0.4692 | |
| WA | 0.586 | 0.9891 | | CA | -0.429 | 0.9447 | |
| CA | 0.594 | 0.9903 | | OR | -0.465 | 0.9595 | |
| OR | 0.597 | 0.9906 | | WA | -0.507 | 0.9732 | |

*MA (latitude: 42.2787, longitude: -71.58) was best with nonlinear regression of $\Psi$ ($r=0.678$, $P_{perm}=0.036$)

Table S8. Signatures of range expansion and origin of USA300-NAE with 15 population dataset (synonymous SNPs).

| Decrease in genetic diversity ($\theta_\pi$) | | | | Increase in derived allele frequency ($\Psi$)* | | | |
|---|---|---|---|---|---|---|---|
| **Origin population** | **r** | **P** | $P_{perm}$ | **Origin population** | **r** | **P** | $P_{perm}$ |
| OH | -0.636 | 0.0054 | 0.073 | PA | 0.493 | 0.0309 | 0.142 |
| PA | -0.618 | 0.0070 | | NY | 0.492 | 0.0313 | |
| NY | -0.609 | 0.0080 | | MA | 0.482 | 0.0346 | |
| MA | -0.590 | 0.0103 | | OH | 0.471 | 0.0383 | |
| SC | -0.477 | 0.0362 | | IL | 0.373 | 0.0855 | |
| IL | -0.443 | 0.0492 | | SC | 0.373 | 0.0857 | |
| GA | -0.401 | 0.0690 | | GA | 0.304 | 0.1353 | |
| FL | -0.312 | 0.1292 | | IA | 0.273 | 0.1625 | |
| IA | -0.270 | 0.1656 | | FL | 0.225 | 0.2106 | |
| MS | -0.204 | 0.2328 | | NE | 0.163 | 0.2810 | |
| NE | 0.024 | 0.5335 | | MS | 0.149 | 0.2979 | |
| TX | 0.222 | 0.7865 | | TX | -0.156 | 0.7099 | |
| OR | 0.414 | 0.9374 | | OR | -0.302 | 0.8628 | |
| WA | 0.421 | 0.9410 | | WA | -0.345 | 0.8961 | |
| CA | 0.474 | 0.9628 | | CA | -0.352 | 0.9007 | |

*Ontario, Canada (latitude: 44.3365, longitude: -76.9676) was best with nonlinear regression of $\Psi$ (r=0.625, $P_{perm}$=0.066)

Table S9. Correlation between *gyr*A resistance allele frequency and estimates of outpatient fluoroquinolone (FQ) use in 15 states.

| **Estimated FQ use in year *** | ***gyrA* resistance allele frequency among** | | | |
|---|---|---|---|---|
| | **All isolates (2001-2011)** | | **Recent isolates (2007-2011)** | |
| | **r** | **P** | **r** | **P** |
| 1999 | -0.126 | 0.6534 | -0.106 | 0.7074 |
| 2009 | -0.326 | 0.2357 | -0.316 | 0.2513 |

Estimates are defined as number of prescriptions per 1000 people (https://resistancemap.cddep.org/CountryPageSub.php?country=United+States)

Table S10. Signatures of range expansion and origin of USA300-NAE with 15 population dataset (*gyrA* SNP excluded).

| **Decrease in genetic diversity ($\theta_\pi$)** | | | | **Increase in derived allele frequency ($\Psi$)*** | | | |
|---|---|---|---|---|---|---|---|
| **Origin population** | **r** | **P** | **$P_{perm}$** | **Origin population** | **r** | **P** | **$P_{perm}$** |
| PA | -0.692 | 0.0021 | 0.025 | MA | 0.546 | 0.0176 | 0.070 |
| MA | -0.690 | 0.0022 | | NY | 0.544 | 0.0180 | |
| NY | -0.690 | 0.0022 | | PA | 0.538 | 0.0193 | |
| OH | -0.679 | 0.0027 | | OH | 0.488 | 0.0324 | |
| SC | -0.552 | 0.0164 | | SC | 0.442 | 0.0495 | |
| GA | -0.484 | 0.0336 | | GA | 0.385 | 0.0782 | |
| IL | -0.427 | 0.0563 | | IL | 0.363 | 0.0918 | |
| FL | -0.398 | 0.0707 | | FL | 0.323 | 0.1204 | |
| MS | -0.259 | 0.1758 | | IA | 0.273 | 0.1628 | |
| IA | -0.235 | 0.1996 | | MS | 0.212 | 0.2238 | |
| NE | 0.100 | 0.6390 | | NE | 0.121 | 0.3338 | |
| TX | 0.126 | 0.6731 | | TX | -0.005 | 0.5068 | |
| WA | 0.565 | 0.9859 | | CA | -0.424 | 0.9425 | |
| OR | 0.571 | 0.9869 | | OR | -0.442 | 0.9506 | |
| CA | 0.583 | 0.9887 | | WA | -0.487 | 0.9672 | |

*MA (latitude: 42.2787, longitude: -71.58) was best with nonlinear regression of $\Psi$ (r=0.688, $P_{perm}$=0.030)

Table S11. Signatures of range expansion and origin of USA300-NAE with 13 population dataset (non-FQ-R Subclade).

| **Decrease in genetic diversity ($\theta_\pi$)** | | | | **Decrease in genetic diversity (tip-to-tip distance)** | | | | **Increase in derived allele frequency ($\Psi$)*** | | | |
|---|---|---|---|---|---|---|---|---|---|---|---|
| **Origin population** | **r** | **P** | **$P_{perm}$** | **Origin population** | **r** | **P** | **$P_{perm}$** | **Origin population** | **r** | **P** | **$P_{perm}$** |
| OH | -0.632 | 0.0102 | 0.103 | OH | -0.633 | 0.0101 | 0.167 | OH | 0.635 | 0.0098 | 0.069 |
| NY | -0.624 | 0.0113 | | PA | -0.586 | 0.0177 | | PA | 0.637 | 0.0110 | |
| PA | -0.622 | 0.0116 | | NY | -0.582 | 0.0185 | | NY | 0.623 | 0.0114 | |
| MA | -0.592 | 0.0166 | | MA | -0.546 | 0.0269 | | MA | 0.603 | 0.0146 | |
| IL | -0.456 | 0.0587 | | IL | -0.534 | 0.0300 | | IL | 0.490 | 0.0447 | |
| IA | -0.367 | 0.1085 | | IA | -0.451 | 0.0608 | | GA | 0.400 | 0.0881 | |
| GA | -0.351 | 0.1196 | | GA | -0.365 | 0.1098 | | IA | 0.397 | 0.0899 | |
| FL | -0.297 | 0.1621 | | MS | -0.332 | 0.1339 | | FL | 0.350 | 0.1203 | |
| MS | -0.238 | 0.2170 | | FL | -0.283 | 0.1749 | | MS | 0.313 | 0.1491 | |
| NE | -0.138 | 0.3265 | | NE | -0.261 | 0.1946 | | NE | 0.125 | 0.3418 | |
| TX | -0.050 | 0.4354 | | TX | -0.150 | 0.3123 | | TX | 0.083 | 0.3941 | |
| CA | 0.398 | 0.9107 | | CA | 0.320 | 0.8569 | | CA | -0.398 | 0.9109 | |
| WA | 0.510 | 0.9624 | | WA | 0.462 | 0.9442 | | WA | -0.616 | 0.9875 | |

*Lake Huron (latitude: 44.3365, longitude: -82.3551) was best with nonlinear regression of $\Psi$ (r=0.629, $P_{perm}$=0.071)

Note: Two of the 15 sampled populations had isolates <4 in the non-FQ-R subclade, so they were excluded from this analysis.

Table S12. Signatures of range expansion and origin of USA300-NAE with 15 population dataset (BratNextGen recombinations excluded).

| **Decrease in genetic diversity ($\theta_\pi$)** | | | | **Decrease in genetic diversity (tip-to-tip distance)** | | | | **Increase in derived allele frequency (Ψ)*** | | | |
|---|---|---|---|---|---|---|---|---|---|---|---|
| **Origin population** | **r** | **P** | **$P_{perm}$** | **Origin population** | **r** | **P** | **$P_{perm}$** | **Origin population** | **r** | **P** | **$P_{perm}$** |
| PA | -0.613 | 0.0076 | 0.086 | OH | -0.599 | 0.0091 | 0.088 | PA | 0.579 | 0.0118 | 0.096 |
| NY | -0.610 | 0.0079 | | PA | -0.599 | 0.0092 | | NY | 0.575 | 0.0124 | |
| MA | -0.606 | 0.0084 | | NY | -0.592 | 0.0100 | | MA | 0.569 | 0.0134 | |
| OH | -0.598 | 0.0093 | | MA | -0.575 | 0.0124 | | OH | 0.554 | 0.0160 | |
| SC | -0.457 | 0.0433 | | SC | -0.436 | 0.0521 | | SC | 0.437 | 0.0519 | |
| GA | -0.391 | 0.0747 | | IL | -0.393 | 0.0737 | | GA | 0.364 | 0.0911 | |
| IL | -0.348 | 0.1019 | | GA | -0.392 | 0.0743 | | IL | 0.310 | 0.1300 | |
| FL | -0.300 | 0.1386 | | FL | -0.303 | 0.1364 | | FL | 0.283 | 0.1531 | |
| IA | -0.178 | 0.2635 | | IA | -0.233 | 0.2020 | | IA | 0.140 | 0.3096 | |
| MS | -0.165 | 0.2789 | | MS | -0.216 | 0.2200 | | MS | 0.128 | 0.3243 | |
| NE | 0.110 | 0.6514 | | NE | 0.030 | 0.5419 | | NE | -0.149 | 0.7021 | |
| TX | 0.170 | 0.7271 | | TX | 0.071 | 0.5989 | | TX | -0.157 | 0.7116 | |
| WA | 0.464 | 0.9594 | | CA | 0.445 | 0.9517 | | OR | -0.446 | 0.9522 | |
| OR | 0.466 | 0.9599 | | OR | 0.447 | 0.9526 | | CA | -0.450 | 0.9538 | |
| CA | 0.495 | 0.9696 | | WA | 0.457 | 0.9568 | | WA | -0.482 | 0.9657 | |

*NY (latitude: 42.4866, longitude: -74.5487) was best with nonlinear regression of Ψ (r=0.599, $P_{perm}$=0.105)

Table S13. Signatures of range expansion and origin of USA300-NAE with 15 population dataset (all recombinations included).

| **Decrease in genetic diversity ($\theta_\pi$)** | | | | **Decrease in genetic diversity (tip-to-tip distance)** | | | | **Increase in derived allele frequency ($\Psi$)*** | | | |
|---|---|---|---|---|---|---|---|---|---|---|---|
| **Origin population** | **r** | **P** | **$P_{perm}$** | **Origin population** | **r** | **P** | **$P_{perm}$** | **Origin population** | **r** | **P** | **$P_{perm}$** |
| FL | -0.737 | 0.0009 | 0.013 | FL | -0.717 | 0.0013 | 0.025 | FL | 0.760 | 0.0005 | 0.04 |
| GA | -0.660 | 0.0037 | | GA | -0.644 | 0.0048 | | SC | 0.704 | 0.0017 | |
| SC | -0.658 | 0.0038 | | SC | -0.629 | 0.0060 | | GA | 0.695 | 0.0020 | |
| MS | -0.559 | 0.0151 | | MS | -0.578 | 0.0121 | | PA | 0.583 | 0.0113 | |
| MA | -0.526 | 0.0220 | | PA | -0.507 | 0.0270 | | MA | 0.582 | 0.0114 | |
| PA | -0.521 | 0.0232 | | MA | -0.503 | 0.0280 | | MS | 0.567 | 0.0138 | |
| NY | -0.505 | 0.0275 | | NY | -0.490 | 0.0318 | | NY | 0.556 | 0.0156 | |
| OH | -0.482 | 0.0345 | | OH | -0.477 | 0.0362 | | OH | 0.538 | 0.0193 | |
| IL | -0.244 | 0.1907 | | IL | -0.268 | 0.1674 | | IL | 0.282 | 0.1540 | |
| TX | -0.026 | 0.4631 | | TX | -0.081 | 0.3868 | | TX | 0.030 | 0.4583 | |
| IA | 0.064 | 0.5891 | | IA | 0.026 | 0.5359 | | IA | -0.050 | 0.5697 | |
| NE | 0.408 | 0.9345 | | NE | 0.349 | 0.8987 | | NE | -0.432 | 0.9462 | |
| CA | 0.683 | 0.9975 | | CA | 0.640 | 0.9949 | | CA | -0.716 | 0.9987 | |
| OR | 0.722 | 0.9988 | | OR | 0.694 | 0.9980 | | OR | -0.766 | 0.9996 | |
| WA | 0.742 | 0.9992 | | WA | 0.720 | 0.9988 | | WA | -0.812 | 0.9999 | |

*FL (latitude: 27.8744, longitude: -82.3551) was best with nonlinear regression of $\Psi$ (r=0. 749, $P_{perm}$=0.01)
